# Sleep-Related Attentional Bias in Insomnia: Time to Examine Moderating Factors?

**DOI:** 10.3389/fpsyg.2018.02573

**Published:** 2018-12-14

**Authors:** Umair Akram, Nicola L. Barclay, Bronwyn Milkins

**Affiliations:** ^1^Department of Psychology, Sociology and Politics, Sheffield Hallam University, Sheffield, United Kingdom; ^2^Nuffield Department of Clinical Neurosciences, Sir William Dunn School of Pathology, Sleep and Circadian Neuroscience Institute, University of Oxford, Oxford, United Kingdom; ^3^Elizabeth Rutherford Memorial Centre for the Advancement of Research on Emotion, University of Western Australia, Perth, WA, Australia

**Keywords:** insomnia, attentional bias, 5HTTLPR polymorphism, worry, misperception

Prominent cognitive models of insomnia have emphasized the notion that the disorder is in part maintained by an attentional bias for sleep related “threat” cues which may be internal (i.e., bodily sensations) or external (i.e., environmental) in nature (Harvey, 2002; Espie et al., 2006). To support this proposition, a growing number of studies have examined the presence of a sleep-related attentional bias for words and images using experimental tasks including the dot-probe, flicker, Posner, emotional Stroop, and eye-tracking paradigms (see Harris et al., 2015 for a review). Many of these studies have provided encouraging evidence for the presence of such a bias in insomnia. However, the evidence base advocating the presence of such a bias remains mixed with a number of studies yielding no statistically significant effects. While a recent review (Harris et al., 2015) cautiously suggests biased attention for sleep-related threat information to be a likely feature of insomnia, the authors highlight the need to understand the specificity of this bias and its relationship with mechanisms believed to underpin the disorder (e.g., sleep preoccupation). Furthermore, whilst it is possible that the mixed evidence may stem from methodological differences relating to the task or population used, the possible moderating influence of these factors on the relationship between attentional bias for sleep-related threat information and insomnia have only recently been examined (e.g., Zheng et al., 2018). With this in mind, we propose candidate factors that may play a crucial role in addressing moderating questions such as “when,” “for whom” and “under which” conditions are sleep-related attentional biases evident in individuals characterized by insomnia.

## 5HTTLPR Polymorphism and Brain Reactivity

First, we consider the role of the serotonin transporter polymorphism (5HTTLPR). Some authors have demonstrated the 5HTTLPR short allele to be related to an increased risk for insomnia/poor sleep quality relative to the long allele (Deuschle et al., 2010; Huang et al., 2014), though findings are inconsistent (e.g., Barclay et al., 2011). Others have documented that the association between 5HTTLPR polymorphism and risk for insomnia/poor sleep is moderated by life stress (Brummett et al., 2007; Huang et al., 2014); and recently the short allele has been associated with increased hypothalamic-pituitary-adrenal (HPA) axis reactivity in response to stress (van Dalfsen and Markus, 2015). However, this association is moderated by sleep quality: long-long homozygotes experiencing poor sleep quality exhibit heightened stress reactivity relative to short-short homozygotes (van Dalfsen and Markus, 2015). Regardless of the mechanisms involved, it appears that 5HTTLPR genotype, sleep quality and stress reactivity are intricately linked, and one possibility is that 5HTTLPR genotype may differentially alter an individual's reactivity to stress under different environmental conditions. In the context of attention bias, it is possible that 5HTTLPR genotype differentially affects behavioral outputs, dependent on sleep quality. In other words, it is possible that 5HTTLPR polymorphisms differentially affect biased attention for “threat” relevant stimuli (possibly due to differences in the activity of the HPA axis) in good vs. poor sleepers.

In other psychiatric populations (e.g. anxiety/depression), a relationship between the short allele and an attentional bias for emotionally salient words and images has been evidenced (Beevers et al., 2009; Fox et al., 2009; Perez-Edgar et al., 2010). Specifically, this allelic variation has been linked with difficulty disengaging attention away from response to threat (Beevers et al., 2009), whereas the long allele has been associated with threat avoidance and increased attention for positively valanced stimuli (Hariri et al., 2002; Munafò et al., 2008). It would be worthy to determine whether a similar pattern would occur in insomnia patients, or whether the presence of poor sleep in this population reverses the 5HTTLPR association as would be expected in light of van Dalfsen and Markus's (2015) finding. Either way, we hypothesize that allelic variation in the 5HTTLPR polymorphism to also modulate the relationship between insomnia and attentional bias.

Given that the amygdala is involved in the processing of emotional information, and because one of the roles of serotonin is regulating mood (Ressler and Nemeroff, 2000), researchers have examined whether 5HTTLPR genotype differentially affects neural responses of the amygdala to emotional stimuli. In healthy participants, short allele carriers exhibit greater neural activity in the amygdala, and greater connectivity between the amygdala and ventromedial prefrontal cortex, compared to long-long homozygotes when presented with negatively toned stimuli (Pezawas et al., 2005; Heinz et al., 2007). Given that individuals with insomnia display increased amygdala reactivity in response to sleep-related stimuli (which may be interpreted as negative; Baglioni et al., 2014) it is plausible that 5HTTLPR genotype may underlie this neural response, with genetically vulnerable individuals exhibiting greater sensitivity, and possibly greater attention, to potential threats.

## Valance of Mood State

Second, previous research has statistically controlled for trait-like emotional distress characterized by disposition to experience anxiety or depression (Jansson-Fröjmark et al., 2012; Akram et al., 2018c). However, attentional bias to threat is a transient process (MacLeod et al., 2002) and emotional tone has differential effects. For example, dysphoric mood is often associated with negative cognitive activity (e.g., catastrophising, worry, rumination) whereas this can be attenuated with enhanced mood (Baglioni et al., 2010, 2014). As such, current mood state should be taken into account in future studies rather than just trait-like characteristics. Indeed, inducing a negative mood state in people with insomnia influences the extent to which an attentional bias to threatening information is displayed (Zheng et al., 2018). Specifically, when assigned to a negative mood inducing (i.e., autobiographical recall of poor sleep) or control (i.e., reading recall) condition prior to completing a dot-probe task comprised of images differing in emotional valence and relatedness to sleep (i.e., general threat, sleep positive, sleep negative), an overall attentional bias to all images emerged only amongst individuals with insomnia who were subject to negative mood state induction Despite an absence of evidence for a sleep-related attentional bias in this study, it remains possible that the overall valance of an individual's emotional mood state, as well as its intensity, duration and frequency of occurrence, may act as moderating factors. That said, further research is required to clarify this position, and expand on these novel findings by examining the therapeutic potential of a positive mood state induction.

## Sleep-Related Worry

Next, we speculate that attentional bias for threatening information might extend particularly to individuals who attribute worry as the main cause of their insomnia. Worry, by definition, is a cognitive process that involves a chain of emotionally negative thoughts about potential future events (Hirsch and Mathews, 2012). Whilst worry may be utilized in a productive manner in terms of solving a specific problem, it appears to be unproductive in the context of sleep, as evidenced by the consistent relationship between worry about sleep and longer sleep onset latency (Harvey, 2002; Harvey and Greenall, 2003; Weise et al., 2013). Indeed, research has consistently evidenced strong relationships between a tendency to worry and high levels of insomnia symptoms (Watts et al., 1994; Jansson and Linton, 2006; Carney et al., 2010; O'Kearney and Pech, 2014). In addition, this population displays a greater propensity to worry during the pre-sleep period, than good sleepers (e.g., Lancee et al., 2017a). Given that by nature, worry is a negative affect-laden process, it is plausible that individuals with insomnia exhibit greater attentional selectivity of emotionally negative information. Therefore, a factor that may moderate this relationship is the degree to which those with insomnia differ in experiencing increased sleep-related worry prior to initiating sleep (e.g., the consequences of not getting enough sleep on functioning; their perceived lack of ability to fix their sleep problem) which is inherently unproductive.

Potentially, a population of those with insomnia who excessively worry about the immediate and longer-term consequences of sleeplessness eventually develop an attentional bias to sleep-related negative information due to an increased personal relevance of this type of information (Espie, 2002). In contrast, those with insomnia who do not worry specifically about sleep are less likely to develop an attention bias to sleep-related negative information. If true, empirical studies would observe a sleep-related attentional bias only in individuals for whom sleep is a major concern on nights where this more profoundly manifested (e.g., attention to signs of wakefulness such as racing heart beat and racing thoughts). While prior research has examined whether cognitive activity in general and worry differentially predict insomnia (Wicklow and Espie, 2000; Harvey and Greenall, 2003), to the best of the author's knowledge no study thus far has examined the specific possibility that differences in the content and intensity of worries (i.e., worry about sleep vs. worry about other topics) differentially predict attentional bias in insomnia. However, prior research has obtained suggestive evidence consistent with this latter possibility in the context of catastrophic worry and sleep-related threat (Barclay and Gregory, 2010; Barclay and Ellis, 2013). In particular, Barclay and Gregory (2010) asked poor and good sleepers to: catastrophise (i.e., iterate negative aspects of a problem: Kendall and Ingram, 1987) about their sleep and a personal worry; and to iterate a hypothetical topic in a positive manner. Here, whilst poor sleepers catastrophised more for each topic compared to good sleepers, the frequency of catastrophic worry didn't vary by topic (i.e., sleep-related worry, personal worry, or hypothetical) for poor sleepers. Additionally, these outcomes were mediated by anxiety. The authors suggest that poor sleepers' orientation of catastrophic thoughts may not always be sleep-specific; rather related to a perseverative iterative style, fuelled by anxiety. Next, Barclay and Ellis (2013) observed that poor sleepers were slower to detect sleep-related stimuli compared to non-sleep-related negative stimuli, and this pattern was not observed in good sleepers. The authors speculate that the personal relevance of the “threat” differentially impacts speed of response: sleep-related stimuli hindered performance, whereas conversely non-specific threats facilitated performance. Taken together, these findings suggest that cognitive styles (i.e., worry focused) and personal relevance of the sleep-related stimuli may moderate the attentional bias effect and may account for the inconsistencies in the literature.

Finally, it is relevant to note that worry and rumination come under the broader cognitive process of “repetitive negative thinking” or “recurrent negative thinking” which is now recognized to be a transdiagnostic cognitive process for anxiety and depressive disorders (Gustavson et al., 2018). First, however we propose examining differential aspects of thought and how this is presented in relation to insomnia and then subsequently determining whether these specific insomnia-relevant constructs moderate the relationship between attentional bias and insomnia. In turn, this area could learn whether the “repetitive negative thinking” phenomenon in anxiety and depressive disorders can be extrapolated to insomnia and other sleep disturbances.

## Misperception of Sleep and Daytime Impairment

It is possible that individuals with insomnia exhibiting a sleep-related attentional bias may be experiencing increased misperception pertaining to their nocturnal sleep and the extent of their daytime impairment resulting from poor sleep. Specifically, it is theorized that increased attention toward sleep-related cues during sleep and the pre-sleep period may distort the distinction between sleep and wakefulness resulting in a misperception of sleep (Harvey, 2002). Indeed, it is well-evidenced that some individuals with insomnia often misperceive attributes of sleep: self-reported sleep onset latency is usually overestimated, and total sleep time underestimated, relative to objectively recorded data (e.g., Wicklow and Espie, 2000; Tang and Harvey, 2006; Van Den Berg et al., 2008). This misperception may also extend to daytime impairment and perception of sleep-deficit. Indeed, whilst individuals with insomnia report their perception of daytime cognitive functioning (i.e., attention, working and episodic memory, problem solving) to be in a manner that confirms the presence of a sleep deficit (i.e., an increased daytime impairment: Fortier-Brochu et al., 2012), objective performance on neuropsychological measures of such functioning do not always coincide with their perception (Orff et al., 2007; Goldman-Mellor et al., 2015). In a similar manner, it has also been evidenced that individuals with insomnia consider their facial appearance to appear more physically tired than they actually are (Akram et al., 2016). However, not all those with insomnia misperceive attributes of their sleep and/or the true extent to which daytime functioning is impaired due to poor sleep. This suggests these two forms of sleep misperception (about sleep and daytime consequences) may be functionally underpinned by a third factor. One plausible factor the present commentary has alluded to is worry. It is possible that worry moderates the relationship between sleep misperception and objective measures of sleep, such that increased levels of worry serve to perpetuate misperception of sleep and of the extent to which poor sleep impairs daily functioning. In contrast, a reduced level of worry may result in more accurate perceptions of one's sleep compared with objective measures. Furthermore, worry and misperception of sleep may facilitate attention toward cues pertaining to nocturnal sleep and daytime performance as a counterproductive form of self-assessment. Here, once attention is placed on a particular cue (e.g., heart rate), it may then be interpreted in a way that confirms the sleep disturbance (e.g., “why is my heart *still* racing” during sleep onset) consequently feeding back to accentuate sleep-related worry (e.g., “If it doesn't slow down soon I won't be able to sleep”) and misperception of sleep attributes (e.g., “I can't function at work because I slept poorly last night”) in a cyclical nature (Harvey, 2002).

## Symptom Variation

Several studies investigating the presence of an attention bias in insomnia have found group differences (e.g., insomnia vs. normal-sleepers) in relation to attentional bias outcomes (Spiegelhalder et al., 2008; Jansson-Fröjmark et al., 2012; Barclay and Ellis, 2013; Beattie et al., 2017; Akram et al., 2018a,b,c; Koranyi et al., 2018). However, increased insomnia symptom severity or severity of poor sleep quality do not appear to be related to attentional bias outcomes (Spiegelhalder et al., 2008; Jansson-Fröjmark et al., 2012; Barclay and Ellis, 2013; Beattie et al., 2017; Akram et al., 2018a,b,c; Koranyi et al., 2018). With that in mind, little variation in yielded effect sizes relating to sleep-related attentional bias in insomnia are reported between clinically diagnosed patients, opportunistic samples of individuals meeting diagnostic criteria, and poor sleepers (Harris et al., 2015). That said, Spiegelhalder et al. (2010) demonstrated positive relationships between attentional bias indices and polysomnographically determined total sleep time, sleep efficiency and duration of slow-wave sleep amongst individuals with insomnia when using the dot-probe task. This pattern of findings did not extend to attentional bias when using the Stroop task. Thus, it is possible that objective measures of severity of sleep disturbance may be predictive of attention bias. Future research should examine the potential role of both subjective and objective variation in sleep continuity as a moderating factor of attention bias.

## Task and Stimuli

There is mixed evidence concerning the presence of a sleep-related attentional bias in insomnia and these inconsistencies may stem from variation in the methodological approach used. Indeed, when examining group differences (insomnia/poor-sleeper vs. control) in reaction time tasks, Harris et al. (2015) determined the flicker, dot-probe and Posner tasks to demonstrate moderate to large effects sizes. In contrast, the Stroop task appears less sensitive, with two studies (Spiegelhalder et al., 2008; Barclay and Ellis, 2013) out of five (Lundh et al., 1997; Spiegelhalder et al., 2008, 2010; Zhou et al., 2018) conducted to date demonstrating an attentional bias in insomnia.

Moving forward from reaction time assessments of attentional bias, which can be considered an indirect measure of attention, a number of recent studies have employed eye-tracking paradigms with the aim to examine selective attention in insomnia (Woods et al., 2013; Beattie et al., 2017; Akram et al., 2018c). Here, visual attention can be continuously recorded throughout stimuli presentation, providing a more ecological observation of visual and selective attention relative to reaction time measures (Armstrong and Olatunji, 2012; Marks et al., 2014). Interestingly, using this methodology, only studies using sleep-related and neutral images, rather than words, as part of a free viewing task evidenced increased attention allocated to the spatial location of insomnia salient stimuli (Beattie et al., 2017; Akram et al., 2018c).

A final consideration is whether people with insomnia compared to normal sleepers differ in the extent to which they consider sleep-related stimuli as *threatening*. Moreover, it remains unclear whether this *threat* drives attentional biases in insomnia; or whether perceptions of threat are stimulated by monitoring of the external environment for sleep-related cues. This latter explanation would be consistent with the idea that the attentional bias in insomnia represents a *craving* for sleep rather than interpreting sleep-related stimuli as *threatening*.

## Application and Summary

Recent evidence from the anxiety literature shows attentional bias modification (ABM) to be effective in ameliorating disorder consistent symptoms amongst individuals who elicit an attentional bias (MacLeod and Grafton, 2016). In poor sleepers, ABM administered immediately prior to bed improved subjective sleep quality and reduced pre-sleep arousal and sleep onset latency across a single sleep episode, relative to alternative nights where a control task was completed (Milkins et al., 2016). Expanding on this research, Lancee et al. (2017b) evidenced no therapeutic effect of ABM amongst those meeting diagnostic criteria for insomnia. As such, the applicability of ABM to insomnia remains elusive. Therefore, studies assessing the efficacy of ABM for insomnia should incorporate measures to assess factors that potentially moderate not only the relationship between attentional bias and insomnia, but also any therapeutic effect. To that end, an ongoing randomized controlled trial of ABM for insomnia is concurrently examining the role of sleep-related worry and sleep-associated monitoring in the therapeutic potential of ABM (Akram et al., 2018a,b). One can postulate that further steps in this line of enquiry would be to (1) determine the mechanism of action of ABM, if successful even in a subset of patients; and (2) identify moderators of response to ABM. Price et al. (2016) have highlighted the inter-relatedness of moderators and mediators in the therapeutic potential of ABM in relation to anxiety. In other words, is the mechanism of anxiety symptom reduction following ABM due to the successful reduction of attention bias (mediator), and is that successful reduction contingent on particular moderating factors? The same questions can be posed in the insomnia arena. It is possible that potential moderators of response to ABM may overlap with those that predict the presence of attention bias, but it is also possible that there may be distinct moderating factors to consider. For example, as suggested in relation to anxiety (Price et al., 2016), potential moderators of therapeutic response to ABM may be strength of attention bias (i.e., positive responders to ABM may be those exhibiting high attention bias); age; ABM training setting (lab vs. home); and clinician assessed outcomes. These are worthy considerations for future trials examining efficacy of ABM in insomnia.

A recent review tentatively supports the notion of a sleep-related attentional bias in insomnia based on six out of nine studies which confirm group differences in relation to attentional allocation to sleep-related stimuli (Harris et al., 2015). However, the number of studies conducted to date still remains limited. Whilst it is possible that a publication bias exists precluding studies demonstrating null effects, to the best of our knowledge, only a further seven studies have been conducted (Woods et al., 2013; Beattie et al., 2017; Akram et al., 2018a,b,c; Koranyi et al., 2018; Zheng et al., 2018; Zhou et al., 2018), of which four provide additional support (Beattie et al., 2017; Akram et al., 2018a,b,c; Koranyi et al., 2018). Therefore, we suggest that further research is required to clarify the presence of a sleep-related attentional bias in insomnia. Additionally, research should pursue the role of potential factors moderating the sleep-related attentional bias/insomnia relationship. In turn, this may allow a particular sub-set of insomnia patients to benefit therapeutically from ABM through appropriate screening.

## Author Contributions

All authors listed have made a substantial, direct and intellectual contribution to the work, and approved it for publication.

### Conflict of Interest Statement

The authors declare that the research was conducted in the absence of any commercial or financial relationships that could be construed as a potential conflict of interest.

## References

[B1] Akram U. Beattie L. Ypsilanti A. Reidy J. Robson A. Chapman A. J.. (2018a). Sleep-related attentional bias for tired faces in insomnia: evidence from a dot-probe paradigm. Behav. Res. Ther. 103, 18–23. 10.1016/j.brat.2018.01.007 29407198

[B2] Akram U. Ellis J. G. Myachykov A. Barclay N. (2016). L. Misperception of tiredness in young adults with Insomnia. J. Sleep. Res. 25, 466–474. 10.1111/jsr.12395 26898988

[B3] Akram U. Milkins B. Ypsilanti A. Reidy J. Lazuras L. Stevenson J.. (2018b). The therapeutic potential of attentional bias modification training for insomnia: study protocol for a randomised controlled trial. Trials 19:567 10.1186/s13063-018-2937-4 30340627 PMC6194703

[B4] Akram U. Robson A. Ypsilanti A. (2018c). Sleep-related attentional bias for faces depicting tiredness in insomnia: evidence from an eye-tracking study. J. Clin. Sleep Med. 14, 959–965. 10.5664/jcsm.7160 29852902 PMC5991958

[B5] Armstrong T. Olatunji B. O. (2012). Eye tracking of attention in the affective disorders: a meta-analytic review and synthesis. Clin. Psych. Rev. 32, 704–723. 10.1016/j.cpr.2012.09.004 23059623 PMC3556338

[B6] Baglioni C. Lombardo C. Bux E. Hansen S. Salveta C. Biello S.. (2010). Psychophysiological reactivity to sleep-related emotional stimuli in primary insomnia. Behav. Res. Ther. 48, 467–475. 10.1016/j.brat.2010.01.008 20227678

[B7] Baglioni C. Spiegelhalder K. Regen W. Feige B. Nissen C. Lombardo C.. (2014). Insomnia disorder is associated with increased amygdala reactivity to insomnia-related stimuli. Sleep 37, 1907–1917. 10.5665/sleep.4240 25325493 PMC4548513

[B8] Barclay N. L. Eley T. C. Mill J. Wong C. C. Zavos H. M. Archer S. N.. (2011). Sleep quality and diurnal preference in a sample of young adults: associations with 5HTTLPR, PER3, and CLOCK 3111. Am. J. Med. Genet. B 156B, 681–690. 10.1002/ajmg.b.31210 21714069

[B9] Barclay N. L. Ellis J. G. (2013). Sleep-related attentional bias in poor verses good sleepers is independent of affective valence. J. Sleep Res. 22, 414–421. 10.1111/jsr.12035 23398166

[B10] Barclay N. L. Gregory A. M. (2010). The presence of a perseverative iterative style in poor vs. good sleepers. J. Behav. Ther. Exp. Psychiatry 41, 18–23. 10.1016/j.jbtep.2009.08.003 19733343

[B11] Beattie L. Bindemann M. Kyle S. D. Biello S. M. (2017). Attention to beds in natural scenes by observers with insomnia symptoms. Behav. Res. Ther. 92, 51–56. 10.1016/j.brat.2017.02.001 28257981

[B12] Beevers C. G. Wells T. T. Ellis A. J. McGeary J. E. (2009). Association of the serotonin transporter gene promoter region (5-HTTLPR) polymorphism with biased attention for emotional stimuli. J. Ab. Psych. 118:670 10.1037/a0016198 PMC2841741 19685963

[B13] Brummett B. H. Krystal A. D. Ashley-Koch A. Kuhn C. M. Züchner S. Siegler I. C.. (2007). Sleep quality varies as a function of 5-HTTLPR genotype and stress. Psychosom. Med. 69, 621–624. 10.1097/PSY.0b013e31814b8de6 17766685 PMC2758820

[B14] Carney C. E. Harris A. L. Moss T. G. Edinger J. D. (2010). Distinguishing rumination from worry in clinical insomnia. Behav. Res. Ther. 48, 540–546. 10.1016/j.brat.2010.03.004 20362977 PMC2871974

[B15] Deuschle M. Schredl M. Schilling C. Wüst S. Frank J. Witt S. H. (2010). Association between a serotonin transporter length polymorphism and primary insomnia. Sleep 33:343 10.1093/sleep/33.3.343 20337192 PMC2831428

[B16] Espie C. A. (2002). Insomnia: conceptual issues in the development, persistence, and treatment of sleep disorder in adults. Annu. Rev. Psychol. 53, 215–243. 10.1146/annurev.psych.53.100901.135243 11752485

[B17] Espie C. A. Broomfield N. M. Macmahon K. A. M. Macphee L. M. Taylor L. M. (2006). The attention-inattention effort pathway in the development of psychophysiologic insomnia: a theoretical review. Sleep Med. Rev. 10, 215–245. 10.1016/j.smrv.2006.03.002 16809056

[B18] Fortier-Brochu É. Beaulieu-Bonneau S. Ivers H. Morin C. M. (2012). Insomnia and daytime cognitive performance: a meta-analysis. Sleep Med. Rev. 16, 83–94. 10.1016/j.smrv.2011.03.008 21636297

[B19] Fox E. Ridgewell A. Ashwin C. (2009). Looking on the bright side: biased attention and the human serotonin transporter gene. Pro. Royal. Soc. Bio. Sci. 276, 1747–1751. 10.1098/rspb.2008.1788 19324793 PMC2674488

[B20] Goldman-Mellor S. Caspi A. Gregory A. M. Harrington H. Poulton R. Moffitt T. E. (2015). Is insomnia associated with deficits in neuropsychological functioning? Evidence from a population-based study. Sleep 38, 623–631. 10.5665/sleep.4584 25348123 PMC4355902

[B21] Gustavson D. E. du Pont A. Whisman M. A. Miyake A. (2018). Evidence for transdiagnostic repetitive negative thinking and its association with rumination, worry, and depression and anxiety symptoms: a commonality analysis. Collabra Psych. 4:13 10.1525/collabra.128 PMC6370308 30761388

[B22] Hariri A. R. Mattay V. S. Tessitore A. Kolachana B. Fera F. Goldman D.. (2002). Serotonin transporter genetic variation and the response of the human amygdala. Science 297, 400–403. 10.1126/science.1071829 12130784

[B23] Harris K. Spiegelhalder K. Espie C. A. MacMahon K. M. A. Woods H. C. Kyle S. (2015). Sleep-related attentional bias in insomnia: a state-of-the-science review. Clin. Psychol. Rev. 42, 16–27. 10.1016/j.cpr.2015.08.001 26284598

[B24] Harvey A. G. (2002). A cognitive model of insomnia. Behav. Res. Ther. 40, 869–893. 10.1016/S0005-7967(01)00061-4 12186352

[B25] Harvey A. G. Greenall E. (2003). Catastrophic worry in primary insomnia. J. Behav. Ther. Exp. Psych. 34, 11–23. 10.1016/S0005-7916(03)00003-X 12763390

[B26] Heinz A. Smolka M. N. Braus D. F. Wrase J. Beck A. Flor H.. (2007). Serotonin transporter genotype (5-HTTLPR): effects of neutral and undefined conditions on amygdala activation. Biol. Psychiatry 61, 1011–1014. 10.1016/j.biopsych.2006.08.019 17157270

[B27] Hirsch C. R. Mathews A. (2012). A cognitive model of pathological worry. Behav. Res. Ther. 50, 636–646. 10.1016/j.brat.2012.06.007 22863541 PMC3444754

[B28] Huang C. Li J. Lu L. Ren X. Li Y. Huang Q.. (2014). Interaction between serotonin transporter gene-linked polymorphic region (5-HTTLPR) and job-related stress in insomnia: a cross-sectional study in Sichuan, China. Sleep Med. 15, 1269–1275. 10.1016/j.sleep.2014.01.023 25154585

[B29] Jansson M. Linton S. J. (2006). The development of insomnia within the first year: a focus on worry. B. J. Health Psych. 11, 501–511. 10.1348/135910705X57412 16870058

[B30] Jansson-Fröjmark M. Bermås M. Kjellén A. (2012). Attentional bias in insomnia: the dot-probe task with pictorial stimuli depicting daytime fatigue/malaise. Cog. Ther. Res. 37, 534–546. 10.1007/s10608-012-9486-z

[B31] Kendall P. C. Ingram R. (1987). The future for cognitive assessment of anxiety: Let's get specific, in Anxiety and Stress Disorders: Cognitive-Behavioral Assessment and Treatment, eds Michelson L. Ascher L. M. (New York, NY: Guilford Press), 89–104.

[B32] Koranyi N. Meinhard M. Bublak P. Witte O. W. Rupprecht S. (2018). Automatic affective responses towards the bed in patients with primary insomnia: evidence for a negativity bias. J. Sleep Res. 27, 215–219. 10.1111/jsr.12591 28833979

[B33] Lancee J. Eisma M. C. van Zanten K. B. Topper M. (2017a). When thinking impairs sleep: trait, daytime and nighttime repetitive thinking in insomnia. Behav. Sleep Med. 15, 53–69. 10.1080/15402002.2015.1083022 26651373

[B34] Lancee J. Yasiney S. L. Brendel R. S. Boffo M. Clarke P. J. F. Salemink E. (2017b). Attentional bias modification training for insomnia: A double-blind placebo controlled randomized trial. PLoS ONE. 12:e0174531. 10.1371/journal.pone.0174531 28423038 PMC5396867

[B35] Lundh L. G. Froding A. Gyllenhammar L. Broman J. E. Hetta J. (1997). Cognitive bias and memory performance in patients with persistent insomnia. Cog Behav. Ther. 26, 27–35.

[B36] MacLeod C. Grafton B. (2016). Anxiety-linked attentional bias and its modification: illustrating the importance of distinguishing processes and procedures in experimental psychopathology research. Behav. Res. Ther. 86, 68–86. 10.1016/j.brat.2016.07.005 27461003

[B37] MacLeod C. Rutherford E. Campbell L. Ebsworthy G. Holker L. (2002). Selective attention and emotional vulnerability: assessing the causal basis of their association through the experimental manipulation of attentional bias. J. Ab. Psych. 111:107 10.1037/0021-843X.111.1.107 11866165

[B38] Marks K. R. Roberts W. Stoops W. W. Pike E. Fillmore M. T. Rush C. R. (2014). Fixation time is a sensitive measure of cocaine cue attentional bias. Addiction 109, 1501–1508. 10.1111/add.12635 24894879 PMC4612370

[B39] Milkins B. Notebaert L. MacLeod C. Clarke P. J. (2016). The potential benefits of targeted attentional bias modification on cognitive arousal and sleep quality in worry-related sleep disturbance. Clin. Psych. Sci. 4, 1015–1027. 10.1177/2167702615626898

[B40] Munafò M. R. Brown S. M. Hariri A. R. (2008). Serotonin transporter (5-HTTLPR) genotype and amygdala activation: a meta-analysis. Bio. Psychiatry 63, 852–857. 10.1016/j.biopsych.2007.08.016 17949693 PMC2755289

[B41] O'Kearney R. Pech M. (2014). General and sleep-specific worry in insomnia. Sleep Bio. Rhyt. 12, 212–215. 10.1111/sbr.12054

[B42] Orff H. J. Drummond S. P. Nowakowski S. Perlis M. L. (2007). Discrepancy between subjective symptomatology and objective neuropsychological performance in insomnia. Sleep 30, 1205–1211. 10.1093/sleep/30.9.1205 17910392 PMC1978394

[B43] Perez-Edgar K. Bar-Haim Y. McDermott J. M. Gorodetsky E. Hodgkinson C. A. Goldman D.. (2010). Variations in the serotonin-transporter gene are associated with attention bias patterns to positive and negative emotion faces. Bio. Psychol. 83, 269–271. 10.1016/j.biopsycho.2009.08.009 19723555 PMC2834856

[B44] Pezawas L. Meyer-Lindenberg A. Drabant E. M. Verchinski B. A. Munoz K. E. Kolachana B. S.. (2005). 5-HTTLPR polymorphism impacts human cingulate-amygdala interactions: a genetic susceptibility mechanism for depression. Nat. Neurosci. 8, 828–834. 10.1038/nn1463 15880108

[B45] Price R. B. Wallace M. Kuckertz J. M. Amir N. Graur S. Cummings L.. (2016). Pooled patient-level meta-analysis of children and adults completing a computer-based anxiety intervention targeting attentional bias. Clin. Psychol. Rev. 50, 37–49. 10.1016/j.cpr.2016.09.009 27693664 PMC5118070

[B46] Ressler K. J. Nemeroff C. B. (2000). Role of serotonergic and noradrenergic systems in the pathophysiology of depression and anxiety disorders. Dep. Anx. 12, 2–19. 10.1002/1520-6394(2000)12:1+<2::AID-DA2>3.0.CO 11098410

[B47] Spiegelhalder K. Espie C. Nissen C. Riemann D. (2008). Sleep-related attentional bias in patients with primary insomnia compared with sleep experts and healthy controls. J. Sleep Res. 17, 191–196. 10.1111/j.1365-2869.2008.00641.x 18482107

[B48] Spiegelhalder K. Kyle S. D. Feige B. Prem M. Nissen C. Espie C. A.. (2010). The impact of sleep-related attentional bias on polysomnographically measured sleep in primary insomnia. Sleep. 33, 107–112. 10.1093/sleep/33.1.107 20120627 PMC2802208

[B49] Tang N. K. Harvey A. G. (2006). Altering misperception of sleep in insomnia: behavioral experiment versus verbal feedback. J. Consult. Clin. Psychol. 74:767 10.1037/0022-006X.74.4.767 16881784

[B50] van Dalfsen J. H. Markus C. R. (2015). Interaction between 5-HTTLPR genotype and cognitive stress vulnerability on sleep quality: effects of sub-chronic tryptophan administration. Int. J. Neuropsychopharmacol. 18:pyu057. 10.1093/ijnp/pyu057 25644221 PMC4360245

[B51] Van Den Berg J. F. Van Rooij F. J. Vos H. Tulen J. H. Hofman A. Miedema H. M.. (2008). Disagreement between subjective and actigraphic measures of sleep duration in a population-based study of elderly persons. J. Sleep Res. 17, 295–302. 10.1111/j.1365-2869.2008.00638.x 18321246

[B52] Watts F. N. Coyle K. East M. P. (1994). The contribution of worry to insomnia. B. J. Clin. Psychol. 33, 211–220. 10.1111/j.2044-8260.1994.tb01115.x 8038740

[B53] Weise S. Ong J. Tesler N. A. Kim S. Roth W. T. (2013). Worried sleep: 24-h monitoring in high and low worriers. Biol. Psychol. 94, 61–70. 10.1016/j.biopsycho.2013.04.009 23643927

[B54] Wicklow A. Espie C. A. (2000). Intrusive thoughts and their relationship to actigraphic measurement of sleep: towards a cognitive model of insomnia. Behav. Res. Ther. 38, 679–693. 10.1016/S0005-7967(99)00136-9 10875190

[B55] Woods H. C. Scheepers C. Ross K. A. Espie C. A. Biello S. M. (2013). What are you looking at? Moving toward an attentional timeline in insomnia: A novel semantic eye tracking study. Sleep 36, 1491–1499. 10.5665/sleep.3042 24082308 PMC3773198

[B56] Zheng S. Feng J. Lin R. Yan Y. Zhang R. Huang H. (2018). The impact of negative mood state on sleep-related attentional bias in insomnia. J. Sleep Res. 23:e12748 10.1111/jsr.12748 30136320

[B57] Zhou N. Zhao C. Yang T. Du S. Yu M. Shen H. (2018). Attentional bias towards sleep-related stimuli in insomnia disorder: a behavioural and ERP study. J. Sleep Res. 27:e12652. 10.1111/jsr.12652 29322673

